# Consumer health technology data in the German healthcare system: Stakeholder perspectives, ethical challenges, and governance pathways

**DOI:** 10.1177/20552076251406307

**Published:** 2025-12-10

**Authors:** Martina Baumann, Maria Maia, Nora Weinberger

**Affiliations:** 1 Institute for Technology Assessment and Systems Analysis, 150232 Karlsruhe Institute of Technology, Karlsruhe, Germany

**Keywords:** Consumer health technology, digital health data, ethics of health data, governance of health research, data solidarity, stakeholder perspectives

## Abstract

**Objectives:**

The paper explores how data generated by consumer health technologies can be used responsibly for research in the German healthcare system. It aims to identify perceived benefits, obstacles, and ethical concerns from different stakeholder groups and to interpret these perspectives through the framework of data solidarity.

**Methods:**

The study employed an explorative qualitative design: data were collected in cross-sector group discussions, semistructured follow-up interviews, or written responses to the interview guide. Stakeholders included healthcare professionals, statutory health insurance representatives, medical researchers, digital health developers, and experts on data governance and ethics.

**Results:**

Stakeholders highlighted several potential benefits of consumer health technology data use, such as patient empowerment, preventive care, advantages for research, and more personalized treatment pathways. At the same time, they pointed to obstacles including lack of interoperability, data quality concerns, and limited trust in commercial actors. Ethical assessments varied depending on the context and purpose of use.

**Conclusion:**

The societal value of consumer health technology data depends on the governance conditions under which it is used. Independent infrastructures, reliable standards for data validity, and practices of solidarity are crucial for realizing benefits while addressing risks. Broader stakeholder involvement and more systematic evaluation will be necessary to strengthen future governance approaches. Besides this, we argue that ethical and societal concerns should not be treated as external constraints of innovation, but as constitutive dimensions of equitable and trustworthy digital health systems.

## Background

Consumer health technologies (CHTs) encompass wearable devices and mobile applications targeting health, lifestyle, and fitness, which are increasingly integrated into daily routines and clinical practices. These technologies collect and process diverse physiological and behavioral data such as heart rate, physical activity, mood, or blood glucose, and are used not only for personal health tracking, but also for prevention, diagnosis, and therapy, or in selected cases, research purposes. In the public discourse, CHTs are often associated with promises of individual empowerment, personalized care, and system-wide efficiency gains.^1,2^ However, their widespread adoption, especially in unsupervised, private and commercially governed contexts, poses a range of risks with regard to data privacy, potential misuse, discrimination of inequitable access, responsibilization of individuals, and erosion of solidarity principles within statutory health insurance, a lack of effectiveness, and potential negative side-effects on health.^1^^,3–6^ These tensions call for an early and context-aware engagement with the ethical and regulatory implications of CHT data practices. While abstract normative debates are important, practical questions, for example, how to design CHT infrastructures and policies that serve public health goals while respecting individual rights, require empirical grounding.

In this article, we examine such questions in the specific context of the German health(care) system, which combines a strong social insurance tradition with emerging frameworks for digital health regulation. In Germany, three categories of CHTs can be distinguished according to their (institutional) context: (1) private or leisure-oriented devices and apps (e.g. fitness trackers), (2) regulated medical applications known as “Digitale Gesundheitsanwendungen” (DiGAs, digital health applications), which are evaluated by the Federal Agency for Drugs and Medical Devices (BfArM), and reimbursed by statutory health insurance,^7,8^ and (3) CHTs embedded in behavioral incentive schemes such as pay-as-you-live tariffs, so called “bonus programs,” where insured individuals receive financial rewards for sharing data and adhering to health-promoting behavior (e.g. taking 10,000 steps a day).^9,10^ Although these technologies are not primarily designed for research, they generate large volumes of health-related data with potential long-term relevance for science and policy. This includes:

- further development of DiGAs and evaluation of CHT effectiveness by using data from the CHT itself,^11^
- using CHT as a tool for clinical studies or health services research,^12,13^
- the dual use of CHTs as both individual disease management tools and aggregated population-level research instruments,^14,15^
- applications in decentralized, user-initiated studies such as during the COVID-19 pandemic,^16^
- and secondary data uses, where data initially collected for personal or commercial purposes are repurposed for research without a predefined goal at the time of collection.^17^

However, realizing this potential faces substantial technical, legal, and societal obstacles. These include challenges related to data access, standardization, representativeness, interoperability, and data quality.^17–22^ Moreover, public trust in CHT-based data donation depends on meaningful transparency, robust governance, and appropriate incentives.^22–24^ Beyond practical concerns, the entanglement between commercial, clinical, and research agendas in the CHT domain raises fundamental ethical questions, for example with regard to the ownership of health data, the accountability of private actors, and the distribution of benefits and risks, particularly in use cases where data generated though public health infrastructure are appropriated for profit.^20,21,25,26^

In Germany, debates have largely focused on DiGA-related implementation barriers, for example inadequate physician remuneration, low adherence rates, and insufficient evidence of effectiveness.^2,27,28^ In contrast, little attention has been paid to the broader societal implications of CHT data use, especially in secondary research contexts. This analytical gap provides the starting point for our study. Our objective is to map the potentials and limitations of CHT data use in Germany from the perspective of those directly involved in or affected by it. To assess the normative implications of our findings, we draw on the concept of data solidarity as proposed by Prainsack et al. This approach offers a framework for evaluating health data practices along three dimensions: (i) fostering beneficial use, (ii) mitigating risks and harm, and (iii) returning profits to the public domain. According to this logic, low-risk data uses with societal value should be actively supported; high-risk applications with limited societal value should be restricted or prohibited and mixed cases, where risks and potential benefits coexist, should be governed through collaborative, context-sensitive mechanisms, for example, redistributive measures like taxation of commercial use or shared governance of data platforms.^29^ Against this background, we address the following questions descriptively from the perspective of stakeholders:

- What types of benefits and potentials are associated with CHT data use in healthcare and research?
- What are the central technical, regulatory, and societal obstacles and challenges for CHT data use?
- Which societal and ethical concerns do stakeholders raise?
- Can distinct use cases be meaningfully categorized by their public value and risk profile?

In a second step, we analyze these findings through the lens of data solidarity by asking:

- How can the conditions for good CHT data use be realized?
- What are the unintended harms and how might they be prevented and mitigated?
- Who benefits from CHT data use and who does not?
- What controversies require public deliberation and stakeholder engagement?

The remainder of the article is structured as follows. We begin by outlining the empirical methodology and presenting key findings along thematic categories. We then interpret these findings in light of normative considerations and close with recommendations for policy and practice.

## Methodology

### Study design and conceptual framework

This study employed an explorative, multimethod approach in order to explore how diverse stakeholders in Germany perceive the opportunities, challenges, and ethical concerns regarding the secondary use of CHT data for research in the German healthcare system. The conceptual lens of data solidarity^29^ guided both the study design and the interpretation of findings. Data solidarity emphasizes conditions under which data practices generate societal value and distribute risks fairly.

### Recruitment and participants

Participants were recruited to capture diverse perspectives from patients, healthcare providers, industry representatives, researchers, and policy stakeholders. Invitations were distributed through professional networks, relevant associations, and direct contacts. Rather than aiming for data saturation or statistical representativeness, the methodological design was oriented toward capturing the heterogeneity, fragmentation, and situated reasoning that characterize the contested and rapidly evolving landscape of CHT data use and governance.

Recruitment combined two main pathways. First, participants for the group discussions were invited in the context of a hybrid stakeholder conference on ethical and societal implications of health data use.^30^ Second, additional participants were identified through purposive sampling based on desktop research. We contacted seven of Germany's largest statutory health insurance providers, 10 practicing physicians, a regional physicians’ association, eight companies developing DiGAs, and 10 researchers working with or on CHTs. Individual CHT users had originally been recruited for interviews focusing inter alia on their personal experiences with CHTs, as well as informed consent preferences. For the present article, however only their contributions to the group discussion were included, as these perspectives could not be separated from the collective setting. At the same time, their views represent a particularly relevant voice in the debate on data use and governance. The interviews themselves addressed different questions and are analyzed and discussed in a separate publication.^31^

For some stakeholder groups, invitations were (also) sent to organizations and not only directly to individuals (e.g. statutory health insurers, regional physicians’ association). In these cases, the number of persons who actually received the invitation is unknown, as distribution was handled internally. This mode is common in exploratory qualitative studies, where access often relies on organizational gatekeepers and snowball-like forwarding processes.^32^ Despite multiple follow-up attempts, the response rate from DiGA manufacturers and practicing physicians remained low, which limited their representation in the final dataset. We interpret this not as a methodological constraint but as a finding itself, potentially reflecting a lack of perceived relevance, institutional openness, or time resources to engage with broader governance and ethics debates around CHT data. However, the overall sample reflects a broad cross-sectoral spectrum of expertise. In total, 27 individuals contributed to the study. To ensure transparency regarding recruitment and sample composition, we provide two complementary tables. Table 1 summarizes the participant flow across stakeholder groups, indicating invitations, responses, and final participation by data collection format. Table 2 details the final composition of participants, including stakeholder roles and the codes that are used throughout the Results section when quoting or paraphrasing contributions.

**Table 1.** Participant flow by stakeholder group.

Stakeholder group	Invitations sent	Responses received	Final participants	Data collection
Medical informaticians	2 (direct contact)	2	2	Written responses
Health insurance representatives	7 organizations contacted, internal distribution unknown	5	5	Written responses, group discussions
Physicians	10 contacted + 1 regional association (internal distribution unknown)	3	1	Interview
Researchers	23 (direct contact)	21	14	Interviews, group discussions
Industry/DiGA	9 companies contacted (internal distribution unknown)	2	2	Written response
CHT users, CHT user/data platform representatives	25+ (direct contacts, social media/snowball method)^a^	4	3	Group discussions, interview
Total	Not systematically recordable	37	27	

DiGA: Digitale Gesundheitsanwendungen; CHT: consumer health technologies.

a See for more details Weinberger et al.^31^

**Table 2.** Participant composition and codes.

Data collection format	Stakeholder description	Code(s)
Written responses	Medical informaticians	MI1, MI2
	Health insurance representatives	K1–K5
	Researchers (medical, sports science)	F2–F5
	DiGA business representative	DiGA1
Interviews, semistructured	Co-founder of health data platform	P1
	Psychologist	F1
	General practitioner	A1
Group discussions	Mixed: researchers (informatics, sociology, sports science, digital health), health insurers, CHT users, quantified self-expert	GD^a^

DiGA: Digitale Gesundheitsanwendungen; CHT: consumer health technologies.

a For the group discussions, contributions were documented under the joint code “GD” rather than assigning individual identifiers, as participants interacted in a collective setting and statements could not always be attributed to single individuals.

### Data collection

Three moderated group discussions with six to seven participants each were conducted. Each session lasted approximately 20–30 min and took place during a project conference. Two of the group discussions were held in an in-person workshop format, where participants documented emerging topics on large sheets of paper and Post-its in a brownbag session style. (Brown bag sessions are informal meetings where people gather during their lunch break to learn from a colleague or guest speaker, who transfers their knowledge and experience about a specific topic to the gathering.) The third group discussion was conducted online, with participants collaboratively mapping concerns and ideas in a digital Miro (Miro is a collaborative online platform for visual work that serves as a digital whiteboard, enabling teams of all sizes to develop ideas, brainstorm, and plan complex projects together, even when they are physically separated. The tool offers an unlimited canvas on which teams can work with templates, notes, mind maps, diagrams, and other content.) board. All group discussions were audio-recorded, and focused on two guiding questions:

1. How are CHT data currently used by different stakeholders (e.g. insurers, researchers, physicians)?
2. How could and should CHT data be used in the future?

Although relatively brief, these discussions were designed to elicit initial cross-sectoral viewpoints and surface discursive tensions rather than to generate full thematic saturation. They primarily served to identify shared concerns, vocabularies, interpretative patterns, and points of contention, which were then explored in more depth in subsequent interviews.

In addition, 16 semistructured interviews were conducted between January and April 2023. To maximize accessibility, participants were offered the choice between a semistructured online interview and answering an open-ended questionnaire via email. The data collection instrument comprised a shared set of guiding questions, supplemented by stakeholder-specific prompts (e.g. regarding bonus programs for insurers; the list of questions is given as the Supplemental materials). Key topics included data protection and consent, current and envisioned data use practices, societal and technical trends, perceived benefits and challenges, and future trends and requirements. Thirteen participants opted for the open-ended written questionnaire format which contained the same guiding questions as the interviews. In cases where initial responses were unclear, incomplete, or raised additional points, follow-up questions were sent by email, involving up to two additional rounds of written exchange. Incomplete answers from one representative of a company with a certified DiGA were also included in the dataset. Three stakeholders decided to participate in the online interview, lasting 30–75 min. These interviews were audio-recorded with participants’ consent, and transcribed verbatim, and subject to qualitative content analysis.

Across all formats, the guiding questions covered data protection and consent, current and envisioned data use practices, societal and technical trends, perceived benefits and challenges, and anticipated requirements for effective and responsible CHT data use. Each method was selected for its specific epistemic function: group discussions revealed shared framings, concerns, and discursive dynamics; interviews offered in-depth exploration of individual reasoning and normative orientations; and written responses allowed for participation by stakeholders less likely to engage in synchronous formats. Triangulating these methods enabled a multiperspective analysis across institutional and professional boundaries.

### Data analysis

For the qualitative content analysis, all team members undertook repeated close readings of the transcripts, written responses, and group discussion notes. This iterative reading process ensured a comprehensive understanding of the material in line with the explorative aims of the study. Emerging patterns, similarities, and differences were documented and subsequently discussed in joint sessions of the research team.

The initial coding scheme was informed by the topics covered in the guiding questions (e.g. such as perceived benefits, challenges, ethical concerns, economic considerations, and envisioned futures). Subcategories were then developed inductively to capture recurrent themes in the empirical material. The process combined independent coding by individual researchers with iterative joint reviews and refinements in team discussions until consensus was reached. The research team comprised members with backgrounds in technology assessment, medical ethics, and science and technology studies, all with prior experience in qualitative research on digital health technologies. This disciplinary and experiential diversity supported analytical transparency and thematic sensitivity.

Reliability was strengthened by the mentioned repeated independent readings, team-based discussions of coding decisions, and adjudication of discrepancies until agreement was reached. The category system was continuously refined during the analysis and is directly visible in the thematic structure of the Results section.

## Results

A total of 29 individuals contributed to the study through written responses (*n* = 13), semistructured interviews (*n* = 3), and group discussions (*n* = 3, with six to seven participants each), whereby six of the 16 participants of the semistructured interview/written response study did also take part in the group discussions.

Analysis of their perspectives revealed four themes and one overarching issue:

1. Opportunities of CHT data use (in healthcare more general): empowerment of patients, preventive healthcare, and more personalized treatment pathways.
2. Technical and organizational barriers (for the use of CHT data in research more specifically): concerns about data validity, lack of interoperability, and missing independent infrastructures.
3. Societal and ethical aspects of CHT use and bonus programs in particular: issues of privacy, commercial exploitation, and uneven distribution of risks and benefits.
4. The envisioned future (with implications for governance orientations): calls for transparent rules, accountability, and trust-building, structured along the three pillars of data solidarity (facilitating good data use (pillar 1); preventing and mitigating harm (pillar 2); and returning profits to the public domain (pillar 3)).^29^

An overarching issue that emerged was divergent stakeholder positions: insurers, researchers, and CHT users emphasized different priorities, with notable gaps in representation from physicians and DiGA developers.

To provide an overview of the empirical material, Table 3 summarizes the main advantages and disadvantages of CHT data use as reported by the stakeholders of our study.

**Table 3.** Reported advantages and disadvantages of CHT data use.

Advantages	Disadvantages/Concerns	Reported by e.g.
Patient empowerment (e.g. counteraction of “learned helplessness,” self-monitoring, autonomy in chronic disease management)	Data privacy and protection concerns, fear of data misuse or stigmatization	Psychologist (F1), insurers (K1), researchers (F5)
Preventive effects (gamification, bonus programs, early diagnosis, lifestyle motivation)	Limited evidence for behavior changes in inactive populations, risk of coercion to prevention	Insurers (K1–K3), group discussants (GD)
Individualization of treatment and counseling; personalized pathways through CHT data and machine learning	Limited validity of some parameters (e.g. sleep quality, energy expenditure)	Researcher (F5), medical informatician (MI1), psychologist (F1)
Improved validity of research data (long-term, contextual, less prone to recall bias)	Lack of interoperability, missing contextual metadata, absence of independent infrastructure	Researchers (F2–F5), medical informaticians (MI2), insurer (K1)
Practical advantages (continuous digital data, reduced administrative burden, novel insights from everyday life)	Unequal access to data across stakeholders, proprietary formats, lack of cooperation	Researchers (F3, F4), co-founder of health platform (P1)
Solidarity and fairness, bonus programs and data donation frames as contributions to collective healthcare	Reinforcement of inequalities (only active/affluent groups represented in data); limited representativeness and generalizability	Insurers (K1–K3), group discussants (GD), psychologist (F1), general practitioner (A1)

CHT: consumer health technologies.

The following sections present these findings in more detail, structured along benefits and potential (“Opportunities of CHT data use”), obstacles and challenges (“Technical and organizational barriers”), societal and ethical aspects (“Societal and ethical aspects of CHT use and bonus programs”), and envisioned future (“The envisioned future and governance”). Only a few selected direct quotations are used to illustrate salient or complex viewpoints, where respondents’ language conveys nuances that would have been lost through paraphrasing. To enhance readability, key findings are highlighted at the beginning of each subsection and supported by illustrative quotes and structured tables summarizing reported barriers and perceived advantages and disadvantages of CHT data use. Where relevant, differences between stakeholder groups are highlighted.

### Opportunities of CHT data use

Stakeholders highlighted substantial benefits of CHT data use, both in healthcare and in research. In healthcare settings, CHT data were seen to promote prevention, empower patients, and support individualized treatment. In research settings, CHT data were valued for their validity, contextual richness, and practical advantages compared with data collected in clinical environments. Many of these benefits depend on the widespread use of CHTs in everyday life, which shapes the conditions for data availability and relevance in secondary research. While these benefits were acknowledged across the board, different stakeholder groups placed emphasis on different aspects: CHT users highlighted empowerment and individualization, researchers focused on validity and contextual richness, and insurers pointed to preventative functions and potential cost savings.

#### Healthcare setting: Prevention, empowerment, and individualization

Stakeholders noted the preventative potential of CHT data, which can motivate users toward healthier behavior, while also supporting monitoring, documentation, and communication between patients and the healthcare system (K1–K3, F1–F3, F5, A1). Examples included gamification and bonus programs promoting behavioral change (K2), tailored feedback on preventative health programs (K1), and early diagnosis of musculoskeletal diseases to prevent unnecessary surgery at a later stage of disease progression (K1). CHT data could also help to identify risk factors, including the influence of living circumstances (F5), and support more holistic anamnesis across the patient journey (K3).

Patient empowerment was emphasized as another important benefit. Transparent access to CHT data may counteract “learned helplessness,” especially in chronic conditions (F1). Quantified feedback can reduce uncertainty in symptom perception, for instance when distinguishing harmless from dangerous causes of dizziness (F1). Respondents highlighted that acute health crises (e.g. hypoglycemia in diabetes) could be prevented through continuous monitoring (F1). Patient-reported outcome measures (PROMs) (A patient-reported outcome is an outcome of a patient's health that is reported directly by the patient who experienced it. This is in contrast to an outcome reported by someone else, such as a physician.) collected by CHTs were also seen to support telemedicine (K3).

Finally, CHT data were seen as enabling more individualized health counseling and treatment, potentially replacing “one-size-fits-all” approaches (MI1, F3, F5, K3, K1). Machine learning was described as a tool to guide the right treatment at the right time, inform patients/the insured about suitable options, and identify subgroups of patients (K3, MI1, K1). Diabetes care was frequently cited: continuous blood glucose monitoring allows acute therapy adjustments and holds potential for automated long-term insulin regulation (F1).

#### Research setting: Improved validity and practical advantages of digital data

Respondents also described multiple research areas where CHT data could be valuable, including motoric, cardiovascular, neurological and psychological diseases, sports medicine, wellbeing, and translational research (F2–F5, MI2). Examples ranged from detecting compensated heart failure by combining activity and clinical data (MI2), to preventing overtraining in sports (F4), or using CHT data as proxy for wellbeing in usability studies with persons with dementia (F5). One respondent also highlighted the potential of CHT data for pediatric research: the movement behavior of children with congenital heart defects could be monitored to inform recommendations for physical exercise frequency and intensity (F5).

Besides this, CHT data were considered to have higher validity compared with data collected in clinical settings (F1–F5, MI1, K3), due to their contextual richness, long-term and continuous measurement, and the authenticity of everyday settings (MI2, F2). Passively collected data were described as less prone to distortion, especially stigma affects self-reporting (F1). Respondents also noted that CHT data could reduce recall bias in PROMs by providing objective control data (MI1). Overall, CHT data were seen to offer a more holistic picture of health, particularly for conditions influenced by daily variability (F5). Practical advantages were also emphasized. CHT data can be collected continuously and digitally, reducing the administrative burden compared with manual forms of data collection (F3, F4). Some respondents gave vivid examples: one described diabetes patients deliberatively testing extreme situations in everyday life, such as “having three glasses of gin and tonic” (F1), which would rarely occur in clinical settings but yields highly relevant data for (exploratory) research.

### Technical and organizational barriers

Stakeholders identified obstacles to the use of CHT data in research that span regulatory, technical, and societal dimensions. They pointed to strict data protection requirements, low interoperability and validity of some parameters, missing contextual information, and the absence of independent infrastructures. In addition, competencies of both users and researchers, limited cooperation between stakeholders, and issues of representativeness and generalizability were seen as critical challenges. In general, the interviews showed that perspectives differed between stakeholder groups: while insurers emphasized data protection and regulatory complexity, researchers stressed validity and interoperability, and CHT users highlighted trust and accessibility.

#### Data protection and regulatory issues

Data protection measures were perceived as essential but demanding and time-consuming, and in specific respects as limiting research (K3, K1, F2, F3, F5, MI2). For example, CHT study devices had to be collected at the end of trials to access locally stored data, making it impossible to view data during the study period (F3, F5). Researchers criticized the lack of high-performance study devices (e.g. limited battery runtime), while privately-owned devices could not be used for research, requiring manual resets between participants (F2). Clinical studies also faced in the respondents’ view additional regulatory hurdles, such as ethics committees requesting full certification of the CHTs as medical devices, which limited device choice and interoperability (MI2). From the perspective of insurer, bureaucratic hurdles and data processing permissions represented further barriers (K1).

Views on data protection itself diverged. One respondent argued that no “revolutionary” new concepts for consent are needed, as existing frameworks are sufficient (MI1). Others emphasized that anonymization, data minimization, and encryption are necessary but challenging in practice (F1, F2, MI2). Concerns were raised about the risk of de-anonymization when linking datasets (GD). Some called for dynamic, context-sensitive consent models, tailored to study participants’ life situation (F1). At the same time, respondents expressed fears of behavioral data being passed to insurers with risks of stigmatization or compliance monitoring (F5). Finally, uncertainties around data ownership, responsibilities, and the legitimacy of broad access to CHT data for research were identified as open questions (GD).

#### Technical data issues

Stakeholders pointed to low interoperability due to missing standards and questioned the validity of certain parameters such as sleep quality or energy expenditure (F2, F4, F5, MI1, K1). Furthermore, contextual information was described as essential but often absent, reducing the search value of CHT data (P1). Linkage of CHTs and clinical data was seen as difficult, both due to lacking standards and the absence of independent infrastructures (MI2). A summary of the technical barriers reported by stakeholders, along with suggested mitigations where mentioned, is provided in Table 4.

**Table 4.** Reported technical barriers and possible mitigations.

Barriers	Reported by e.g.	Proposed mitigations
Low interoperability due to missing standards	Researchers (F2, F4, F5), medical informatician (MI1), insurer (K1)	Development of common standards; integration with clinical infrastructures
Limited validity of certain parameters (e.g. sleep quality, energy expenditure)	Researchers (F2, F3, F5), medical informatician (MI1)	Validation, studies; long-term accuracy testing
Missing contextual information (e.g. environment, lifestyle factors)	Platform developer (P1)	Enrich CHT data with contextual metadata
Lack of independent infrastructure for CHT-clinical data linkage	Medical informatician (MI2)	Establish non-commercial, independent data platforms
Device certification requirements (clinical studies requiring full certification as medical device)	Medical informatician (MI2)	Regulatory clarification; adaptation of ethics committee procedures
Limited battery runtime and hardware performance of study devices	Researchers (F2, F3, F5)	Development of research-grade devices
Restricted reuse of privately-owned devices in research	Researcher (F2)	Policy change or certification pathways for consumer devices

CHT: consumer health technologies.

#### Competencies needed for the use of CHTs

Validity of data was also considered dependent on the correct use of the device by patients, medical staff, and study participants (MI1). Respondents noted that CHT users need a certain degree of technology affinity, and certain groups, for example elderly patients, may face challenges (F2, A1). Clear communication of device limitations and potential errors (“onboarding”) was regarded as essential to avoid false expectations leading to frustration, rejection, or harm (F1). Several respondents emphasized that unclear protocols or incorrect handling, such as carrying a device in the wrong pocket, can easily compromise data quality (F2, P1). Close researcher–study participant interaction was seen as beneficial, suggesting that smaller-scale studies can be more effective than large ones (P1).

#### Data access and availability, willingness to share data, and cooperation between stakeholders

Access to CHT data was considered highly uneven. Device manufacturers have the best access to data in terms of participant numbers (P1), but rarely share data with clinicians or researchers (MI1), though access to DiGA data could sometimes be given upon request (DiGA1). Users themselves have difficulties to access and reuse their data due to proprietary formats (F1). Clinicians cited lack of time, expertise, and incentives to engage with patient data (MI1), and insurers are restricted to their own bonus program data (GD). Respondents called for equal and controlled access to CHT data for all stakeholders with legitimate interests.

Motivations for data sharing were also debated. Users’ willingness to donate data was often constrained by mistrust, especially if insurers are the recipients (K1). Respondents questioned the fairness of expecting patients in poor health or with limited resources to contribute data for research (P1). While financial incentives were seen as problematic and potentially leading to “fake” data (P1), involving patients in research design (e.g. choice of research questions) was considered a promising alternative to foster engagement and relevance for patients’ daily lives (P1). Besides this, respondents highlighted a lack of cooperation not only between stakeholders but also across (scientific) disciplines, such as informatics and behavioral science, which hinders the design of meaningful projects (F2).

#### Data interpretation, representativeness, and generalizability

Stakeholders noted that CHT data require careful methodological consideration to be valuable (GD). Researchers, insurers, and the general practitioner emphasized the need for analytic competencies and support by clinicians with CHT experience (F2, K1, A1). Furthermore, respondents pointed to challenges in reducing and visualizing complex datasets (F5, P1) and underlined the importance of contextual knowledge and reference values for complex datasets (F5). Reference data also has to be communicated carefully to avoid distress in patients not within “normal” ranges (F1). Concerns were also raised about longitudinal comparability, as device manufactures frequently update algorithms in ways not transparent to researchers and users (P1). Study's participants further noted that motivation in bonus programs may fade quickly, limiting the value of short-term studies (K2).

In addition, representativeness was questioned. CHTs are often used by younger, more affluent, or already active individuals (A1, F1, K1, K2). As a result, CHT data may systematically underrepresent older, less affluent, or chronically ill populations. While some respondents argued that single-person studies can still yield insights (P1), others stressed that contextual factors make CHT data highly individualized, limiting generalizability (A1).

### Societal and ethical aspects of CHT use and bonus programs

Stakeholders expressed ambivalent views on the societal and ethical implications of CHT and bonus programs. Criticism centered in limited evidence for behavioral change, the risk of reinforcing inequalities, and tensions between empowerment and coercion. At the same time, not surprisingly, insurers defended bonus programs as fair, solidarity-enhancing, and potentially cost-saving. A further theme concerned the information needs of data donors, with calls for transparent, accessible communication about data protection, rights, and benefits.

#### Critique of and uncertainties regarding bonus programs

Insurance representatives acknowledged weaknesses of bonus programs. Internal monitoring suggests a bandwagon effect: already physically active individuals take part in bonus programs that reward this behavior (K1), while little evidence indicates that less active persons are motivated to change behavior (K1). Insurance providers also admitted that healthy behavior is more complex than current bonus apps can capture (K2, K3). Moreover, bonus programs are sometimes used strategically to attract healthier, more health-oriented populations, such as craft workers. Group discussants were critical of a general push toward preventative CHT use and bonus apps in particular. They questioned the cultural and normative framing of prevention, debated the meaning of solidarity in healthcare, and highlighted the socioeconomic component of health. A fine line was identified between self-empowerment and coercion toward prevention (GD).

#### Defense arguments for bonus programs

Despite criticism, respondents also emphasized the positive functions of CHTs and bonus programs. They were seen as raising awareness about lifestyle effects and strengthen self-care as an element of solidarity (GD). Insurers argued that bonus programs:

- help reduce migration to private insurers, thereby supporting the solidarity principle (K1);
- are capped by law at 600 euros annually and do not restrict access to healthcare based on behavior (K1, K3);
- are open to all insured persons in principle (K3, K1); and
- reward individual initiative while contributing to cost savings (K2, K3).

Health economic benefits of CHTs (DiGAs) have not yet been demonstrated, as systematic evaluations are typically required only after 3 years in the German statutory system (K1). However, the same health insurance representative suggested that positive effects are nonetheless expected, as CHTs (DiGAs) would not be available on prescription otherwise (K1). Evaluations of bonus programs by insurers are not publicly available; one study conducted by an independent institute and reported to the Federal Ministry of Labor and Social Affairs focused according to one insurer only on cost savings, not on health outcomes (K3).

#### Information needs of data donors

Insurance representatives emphasized that they collect only sparse data through bonus apps (e.g. app usage for mindfulness or fitness) and would not forward such data to third parties. Data protection was framed as trust-building measure that needed to be communicated clearly, transparently, and in accessible formats (MI1, K2, K3).

Respondents described highly diverse user attitudes, ranging from “no worries at all” to deep skepticism about whether uploaded data would remain private (P1). According to the stakeholders, one explanation for this diversity is the varying levels of information about data protection measures. Key aspects that should be communicated included the purpose of data collection, the location of the server, storage duration, and the right/option to withdraw consent (K2, K3). Principles such as data minimization should be communicated, for example clarifying that bonus programs collect only app usage data, not vital parameters, and are not shared with third parties (K1, K2). Motivating insured persons and patients to donate data was seen as requiring more than legal frameworks. The stakeholders argued that emphasizing data donation as an act of solidarity could encourage participation by showing its contribution to collective healthcare and stable insurance premiums (K1). Beyond data frameworks, respondents called for transparency about how data would be analyzed, what results would be produced, and how these affect healthcare systems and users’/patients’ daily lives (F5, GD).

### The envisioned future and governance

Respondents regarded the current use of CHT data in Germany as still at an early stage, with limited projects and slow progress in digitalization. Wishes and requirements expressed for the future were modest, focusing on building trust, establishing technical standards and infrastructures, and clarifying regulatory responsibilities. In general, the results show that in some points the stakeholders’ perspectives diverged. Insurers were cautious and emphasized restricted access and trust-building. Researchers highlighted the need for secure and independent infrastructures as well as validation of data quality, while group discussants called for broader societal debates on solidarity, ownership, and the purposes of CHT data use. Looking forward, three main drivers of CHT data use were identified: growing demand from patients and insured persons, technical advances in data quality and algorithms, and economic pressures in the healthcare system.

#### Situation regarding CHT data use

The responses conveyed the impression that CHT data use is still at an early stage. In many areas, the question of what should or can be done with the data remains unresolved (F5). Several pointed to the slow pace of digitalization in German healthcare and expressed skepticism about the speed of future progress (K1, K3, DiGA1). Insurance representatives reported no planned projects and were uncertain whether a single insurer could initiate research alone or whether coordination at the level of the statutory health insurance system (Gesetzliche Krankenversicherung) would be necessary (K1). Envisioned applications of CHT data included research on prevention (K2), and improved diagnosis and therapy via telemedicine using AI combined with CHTs and electronic patient record (ePA) data (K3). The integration of CHT data into the ePA was widely considered inevitable sooner or later (K3, DiGA1). However, synergies from combining digital data from diverse sources for research have so far not been exploited (DiGA1).

#### Wishes and requirements

The wishes expressed were relatively modest and addressed societal (trust and engagement), technical (data standards and platforms), and regulatory (overregulation through medical device regulation) issues. Some insurance providers described themselves as disadvantaged due to restricted access to health data (GD, K4), but also expressed optimism:

…that the broad establishment of the electronic patient record will reduce reservations and contribute to greater trust, especially when it comes to the handling of data by health insurance companies. People will experience the advantages of better data use in very concrete terms and will then also demand that more. (K4)

Group discussants called for a public debate on legitimate purposes of CHT data use, ownership, and consent models, as well as the establishment of use and access committees similar to those already applied for clinical data. They emphasized that many fundamental questions regarding responsibilities remain unanswered, and highlighted a lack of research on the intrusiveness of lifestyle-based recommendations (GD). Researchers emphasized the need for secure and sustainable infrastructures. While some argued for a central data platform despite higher security risks (e.g. F1), others preferred decentralized platforms being developed and supported by long-term funding (P1). Views on commercial players were also mixed: while some respondents stressed mistrust in large corporations (like Google) and preferred independence (MI1, K1, K2), others saw potential synergies in collaborations between commercial players and statutory insurers (e.g. K3). The broader establishment of the ePA was generally regarded as a prerequisite for broader CHT integration, through active engagement by clinicians and patients was seen as a major obstacle. One interviewee envisioned a specialist doctor for data-driven medicine to address this challenge (MI1).

Basic requirement for CHT included data standards (which are in principle already prescribed by the BfArM for DiGAs) (P1, F1, MI2, MI1), longitudinal data and reference values for interpretation (F5), and validation of measurements and recommendations. Several participants warned against comparing individual values to population-wide norms, which might be misleading or harmful (A1, P1, F1). A sports scientist illustrated risks of unvalidated consumer products:

Studies show, for example, that people feel weaker and less rested when their smartwatch shows them poor sleep quality (even if this is only suggested and people actually slept well). Especially in the sports and health market, new products are pushing their way at a rapid pace, which often do not keep what they promise. While the duration of sleep can now be recorded relatively accurately, this has not been the case for the sleep quality parameter so far, but is suggested by numerous products. There is therefore a potential risk that wearables will issue false data and recommendations, which will be implemented accordingly by the user and become a risk in terms of health and performance. (F4)

One researcher saw the medical device regulation as an innovation obstacle for smaller businesses, while at the same time not providing a sufficient mechanism to control the quality of devices of big companies which have the financial means to conduct their own studies (F1).

#### Future trends and drivers of CHT use

The respondents pointed out three main drivers. First, demand from patients and insured persons is growing, fueled by fitness and lifestyle trends, pandemic experiences, and increasing health awareness. Respondents noted that many want their data to be used meaningfully, for example by sharing it with sports physicians or interpreting raw data themselves (K2, K3, MI1, F4, GD). Second, technical progress was seen as a driver, particularly in developing transparent algorithms, automatic non-invasive data collection, and more valid and reliable parameters (F4, MI2, F2, F1, F5, GD). Third, economic pressures in the healthcare system were described as a force pushing toward prevention and data-driven care (F1, K1). Some respondents suggested that digital health applications remain lucrative in Germany, and that disease definitions might shift toward preclinical states, increasing the target population (F1).

Societal and ethical aspects were seen as neither driving nor inhibiting, but as a steering force for technological developments such as artificial intelligence in health and CHT use (F1). Potentially inhibiting factors were also pointed out: the risk of data misuse (MI2) and a restrictive interpretation of data protection, as one respondent puts it:

…data protection is often used as a final argument when a procedure should or may not proceed. This is then followed by a reflex demand and statement that data protection prevents research…. (MI1)

Finally, a respondent warned of a passive attitude among stakeholders of the German healthcare system, which could result in leaving the field to commercial actors (K1).

## Discussion

This study explored stakeholder perspectives on the potential and challenges of using CHT data for research in Germany. Our findings highlight substantial opportunities, including patient empowerment, preventive health, individualization of treatment, and improved validity of research data. These expectations echo broader debates in the digital health field.^1,21,22^ At the same time, respondents emphasized structural, technical, and ethical barriers such as regulatory burdens, limited interoperability, data quality concerns, and unequal access across stakeholders. While international studies have emphasized additional benefits such as reduced infrastructure costs and efficiency in recruitment and screening,^25,33^ these were largely absent from our material, reflecting the German context in which privately owned devices cannot be used for research due to strict data protection rules. The real-world impact of digital health applications also remains contested, with recent evaluations suggesting limited effectiveness and cost-efficiency.^7,11^ In sum, CHT data were described as a double-edged phenomenon: rich in potential but strongly dependent on governance frameworks and infrastructures.

### Interpreting benefits and challenges through the lens of data solidarity

The framework of *data solidarity*^29^ provides a useful perspective for interpreting these findings. It highlights that data use should be supported in cases of high public value and low risk (pillar 1), harm mitigation instruments should be in place in cases of high public value and high risk (pillar 2), and commercial profits resulting from data use should be shared with the public (pillar 3).

Overall, the societal value of data practices depends on the fair distribution of risks and burdens. Our results conform this, while also revealing tensions in how solidarity is enacted.

- The first pillar, fostering beneficial use, depends highly on data *availability and access*. According to our stakeholders, it is reflected in the wide collection of CHT data alongside fragmented and uneven access. This resonates with earlier work emphasizing proprietary data silos and companies’ reluctance to support secondary data use without commercial gain.^17,34^ Although some surveys suggest researchers do not always view access restrictions as the main obstacle,^19^ our findings indicate that the absence of a common infrastructure remains a barrier. The ePA (German electronic patient record) was repeatedly cited as a possible solution, nut its slow rollout due to societal reservations and legal complexities limits its potential.^35,36^
- The second pillar, harm prevention and mitigation in high-risk data use cases, depends highly on data *quality and validity.* Wearables and related applications are predominantly used by younger, affluent, and health-literate populations,^25,28^ while disadvantaged groups remain underrepresented. This concern was also mentioned by participants in our study and challenges claims about the representativeness and inclusiveness of digital health data. The stakeholders mentioned also concerns about the accuracy of parameters such as sleep quality. Similar doubts are echoed in the literature on consumer health metrics and their effects on users.^6,28^
- The third pillar, returning profits to the public domain, is highly linked to aspects of *solidarity and trust* and was reflected in the conditional framing of data donation. Participants emphasized that data sharing requires robust protection, transparency, reciprocity, and participation of data donors in decision-making processes, a finding consistent with earlier studies of health data practices.^31^ The ambivalence of solidarity became visible, for example, in discussions of bonus programs, where empowerment could blur into coercion. Such tensions mirror wider debates about conditionality in welfare systems.^3,31^

Taken together, these examples show how the three pillars of data solidarity can be operationalized as assessment criteria: equitable access across stakeholders, validated and meaningful data quality, and governance practices that safeguard trust and solidarity. Applying these criteria reveals why CHT data cannot be considered inherently beneficial or harmful, but only in relation to the governance conditions that shape their use.

### Societal and ethical implications

Bonus programs crystallize the ambivalences with regard to ethical and societal implications of CHT data use. While insurers portray them as solidarity-enhancing and patient-empowering instruments, capped by all and formally open to all, evaluations of their health benefits remain scarce. Available studies and our findings indicate that preventive effects of CHT may be limited and that such programs may largely benefit already active groups.^7,11,37^ Concerns that they may undermine solidarity by privileging healthier subpopulations mirror broader critiques of digital health interventions, particularly regarding their potential to reinforce inequalities.^38,39^

Physicians also express doubts regarding the (cost-)effectiveness of CHTs compared with other therapeutic interventions,^37^ ultimately raising ethical concerns about the just allocation of healthcare resources. The lack of robust evidence has been highlighted by both independent evaluations and methodological analyses pointing to the difficulties of evaluating CHTs in real-world contexts.^40^ The Institute for Quality and Efficiency in Health Care^41^ similarly concluded that the current evidence base does not allow robust conclusions. Consequently, the use of CHT data raises not only concerns about the just distribution of benefits and risks across subpopulations, but also about the balance between public benefits and corporate profits from app development. This system-level aspect—which may be less apparent to individual patients participating as data donors—should be prioritized to the same extent as safeguarding trust and solidarity, two values emphasized by our participants.

### Governance and policy implications

Several governance priorities follow from these findings. First, independent infrastructures are needed to overcome asymmetries of access and reduce dependence on commercial actors, a demand echoed by previous research on digital health platforms.^17,34^ The German ePA was referred to by the respondents several times as a potential future platform for CHT data collection, yet its nationwide implementation is slow and its usability limited due to societal reservations, practical barriers, and legal complexities.^35,36^ A look at other countries demonstrates that successful solutions for managing and utilizing electronic health data already exist. Finland has implemented a comprehensive national system for electronic health data, known as Kanta, complemented by a dedicated legal and organizational framework for the secondary use of health data through the authority Findata.^42^ The Finnish model extends beyond mere clinical documentation, emphasizing data accessibility for authorized research and innovation purposes. Denmark is recognized as a leader in digital healthcare, having implemented extensive digital solutions early on, such as the electronic health records (EHRs) and the National eHealth Platform. A practical example is the Sundhed.dk platform that was launched in 2003 allowing citizens to access their health records, medication information, and laboratory results, facilitating better patient engagement and self-management. Denmark has established a robust infrastructure for data sharing among healthcare providers, which enhances patient safety and treatment outcomes.^43,44^

The key lessons for other countries, including Germany, are twofold: first, secondary use of health data for research should be integrated into the design of EHR systems from the outset; and second, robust legal, ethical, and governance structures are as crucial as technical interoperability for ensuring trust, efficiency, and societal benefit.

Second, systematic validation of CHT data and collection of real-world evidence on CHT effects^45^ is essential for research reliability and to protect users from harmful or misleading feedback.^6^ Existing proposals to address these risks include platforms for independent quality checks,^46^ certification schemes such as the DiGA process, and participatory development of CHTs with vulnerable groups.^47^ Privacy literacy also remains uneven, and gamified or more accessible communication of data protection rules has been proposed to increase user awareness.^48^

Third, stakeholder cooperation requires further strengthening, although initiatives such as the European Union's eHealth Stakeholder Group already bring together patients, providers, researchers, and industry to advise on policy and interoperability.^49^ Particularly across disciplinary boundaries, ineffective collaboration has been identified as a recurring barrier for CHT data use in both our study and other analyses, suggesting the need for more inter- and transdisciplinary research in digital health.^28,34^

Participatory design approaches provide important models for addressing gaps in CHT accessibility and diminish the risk of reinforcing inequalities.^39,47,50^ Furthermore, broader public debate on the legitimate purposes of CHT data use is crucial. The contentious discussions on secondary use of clinical data in Germany^51,52^ demonstrate the need for transparent mechanisms for CHT governance, such as use and access committees, ideally including patients. Estonia, for example, gives citizens direct access to their medical records via a patient portal, allows them to see who has accessed their data, and involves citizens in decision-making around the digital health system.^53^

Data ownership and informed consent are widely recognized as key ethical and legal obstacles to the sharing of CHT data.^17^ Participants in our study echoed these concerns, particularly regarding the incentives for data sharing. Some viewed monetary incentives as problematic, potentially exacerbating inequities if only certain groups are able to contribute data during the periods of acute illness. The commercial use of health data raises additional challenges: a recent proposal has advocated for fair price negotiations when companies profit from publicly funded clinical data.^54^ In the context of digital health applications, such negotiations partially account for user-generated data contributions^7^; nevertheless, patients’ roles as data contributors are rarely acknowledged in these discussions.

## Conclusion

This study explored the conditions under which CHT data can be used responsibly for research within the German healthcare system. Drawing on diverse stakeholder perspectives, it mapped both expected benefits and the structural, technical, and normative tensions of current data governance. Using the concept of data solidarity as an interpretive lens, the analysis showed that responsible CHT data use requires enabling beneficial practices, mitigating risks, and distributing benefits fairly.

**Figure 1.**
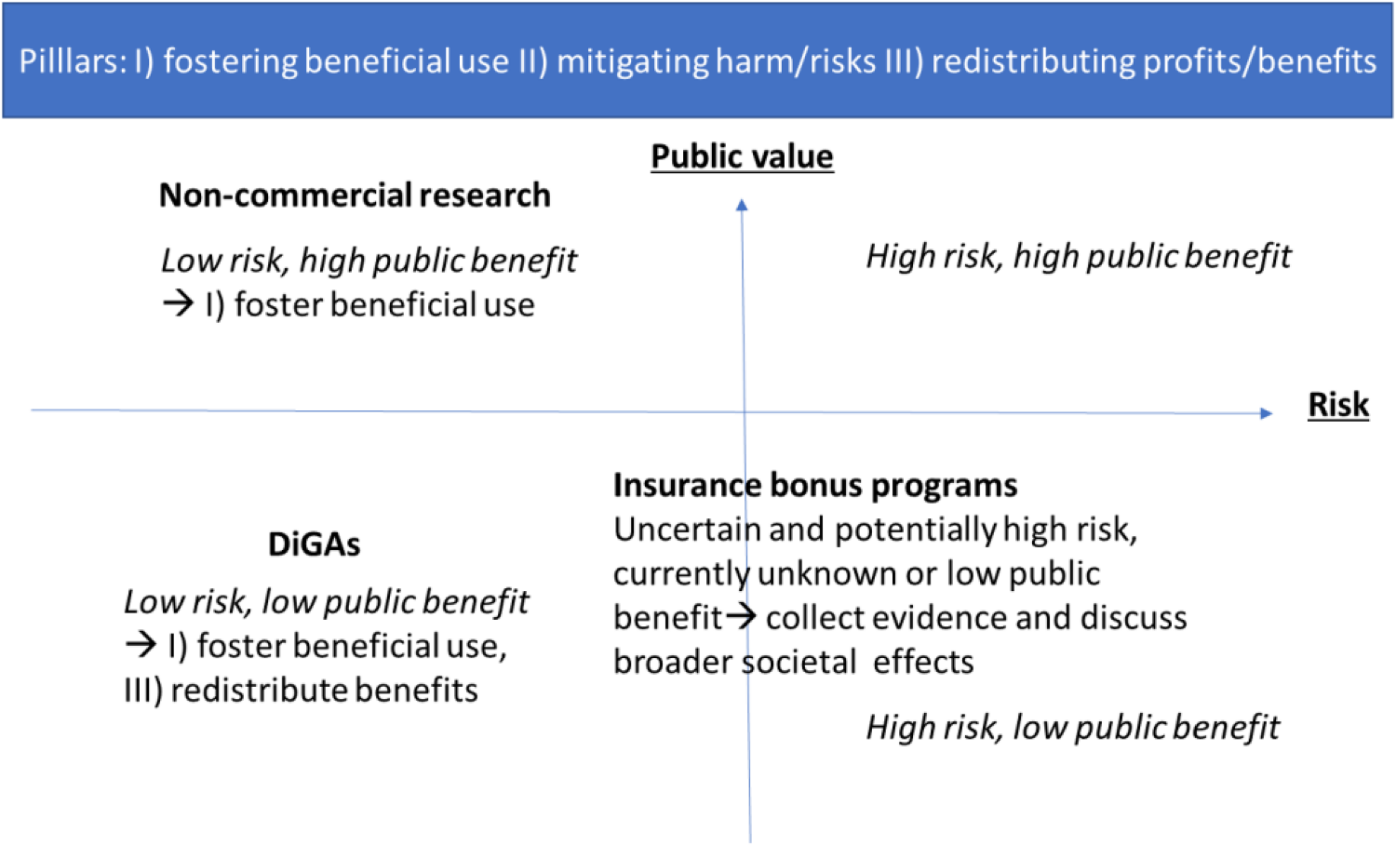
Different use cases for CHT data based on risk and public value. Source: adapted from Prainsack et al.^29^ CHT: consumer health technologies.

The findings suggest that the public value of CHT data use depends strongly on the context and purpose (see Figure 1).

Non-commercial research, conducted by academic or public institutions, is widely perceived as high in public value with comparatively low risk when accompanied by robust safeguards. DiGAs represent a more ambiguous case: while they hold potential for innovation and care improvement, their benefits remain difficult to assess given limited evidence, commercial interests, and restrictions on data access. Bonus programs, by contrast, emerged as a low-value, high-risk case, raising particular concerns about privacy, data misuse, and the erosion of solidarity.

Responsible CHT governance must therefore be guided by ethical principles of inclusiveness, fair benefit distribution, respect for autonomy, and careful balancing of privacy protection and feasibility. Regulation should require demonstrable public value as a condition for CHT deployment and ensure that price negotiations for commercial products reflect both effectiveness and fair profit sharing. Independent, high-quality data infrastructures, whether drawing on DiGA data or publicly governed platforms, are essential to support non-commercial research that serves the common good. Societal and ethical considerations should not be understood as barriers but as guiding forces for responsible innovation. Transparent communication among stakeholders and open public debate on benefits and potential negative side-effects are key for building trust. Beyond these normative implications, our findings underline the importance of integrating ethical foresight and governance reflexivity into the design, evaluation, and deployment of digital health technologies, especially where data is repurposed for public value. Further research is needed to evaluate the long-term societal effects of CHT-based incentive programs, examine user perspectives in more diverse and vulnerable populations, and compare international regulatory and ethical frameworks for secondary data uses.

### Limitations of the study

This study was designed as a small-scale study, following an explorative qualitative design with purposive recruitment and does not claim statistical representativeness. Its contribution lies in mapping heterogeneous perspectives and identifying tensions and governance challenges, rather than in producing generalizable conclusion. While a broad range of stakeholder perspectives was included, some voices were underrepresented despite repeated recruitment efforts, particularly practicing physicians and DiGA manufacturers. This underrepresentation may itself be interpreted as an empirical finding, pointing to limited willingness or capacity of these stakeholders to engage in broader debates on CHT governance. Furthermore, member checking with participants was not conducted. While this is not uncommon in expert-based studies, it may have constrained opportunities to validate interpretations with participants. Finally, the study relied primarily on qualitative accounts, triangulation with independent evaluative data was beyond its scope. These limitations should be considered when interpreting the findings.

## Supplemental Material

sj-docx-1-dhj-10.1177_20552076251406307 - Supplemental material for Consumer health technology data in the German healthcare system: Stakeholder perspectives, ethical challenges, and governance pathways Supplemental material, sj-docx-1-dhj-10.1177_20552076251406307 for Consumer health technology data in the German healthcare system: Stakeholder perspectives, ethical challenges, and governance pathways by Martina Baumann, Maria Maia and Nora Weinberger in DIGITAL HEALTH

sj-pdf-2-dhj-10.1177_20552076251406307 - Supplemental material for Consumer health technology data in the German healthcare system: Stakeholder perspectives, ethical challenges, and governance pathways Supplemental material, sj-pdf-2-dhj-10.1177_20552076251406307 for Consumer health technology data in the German healthcare system: Stakeholder perspectives, ethical challenges, and governance pathways by Martina Baumann, Maria Maia and Nora Weinberger in DIGITAL HEALTH
