# Supplementary material for: Consumer health technology data in the German healthcare system: Stakeholder perspectives, ethical challenges, and governance pathways
Source: Digit Health. 2025 Dec 10;11:20552076251406307. doi: 10.1177/20552076251406307 (PMC12696318; doi:10.1177/20552076251406307)
Supplement: sj-docx-1-dhj-10.1177_20552076251406307 - Supplemental material for Consumer health technology data in the German healthcare system: Stakeholder perspectives, ethical challenges, and governance pathways [file sj-docx-1-dhj-10.1177_20552076251406307.docx]

**Common questions (with specific formulations fitting the field of the expert)**

- Do you use CHT yourself in your private life, and if yes, why?
- Which benefits and potential of CHT do you see in your professional field/work? (please describe an example of a successful use case, if possible)
- Which hurdles, challenges and risks do you experience when using CHT in your professional field/work?
- Which novel developments in the area of CHT do you expect that could influence your professional field/work?
- Which societal trends do you observe or expect that will influence CHT (data) use?

**Additional, expert specific questions:**

Physicians

- How well informed do you feel about CHTs?

DIGA companies

- Could you please describe in more detail (by way of example) which data is collected, stored, displayed and interpreted by the app and how?
- What data protection concepts do you work with? (What are the particular challenges here with regard to CHTs?)
- What form of consent do you work with? What challenges and opportunities do you see here with CHTs?
- How realistic do you think the integration of CHT data with other types of data (health insurance billing data, care data, etc.) is?

Health insurance representatives

- In your opinion, what is important when a health insurer as a data recipient communicates the purpose of data use to potential data donors (insured persons)?
- What effects do you hope to see from bonus programmes in which policyholders collect bonuses by using a fitness app? What is known about the target group-specific effects of bonus programmes so far (is there any internal research on this)?
- What do you think of the criticism that the idea of solidarity would be devalued by the establishment of bonus programmes or that there could be unfair treatment of insured persons (misuse of data or unequal opportunities to participate in bonus programmes), or that the bonus programmes do not make sense because health behaviour is too complex to be recorded in a bonus app?
- (Why) do you think it would be desirable for health insurers to have a data infrastructure for CHT data that is independent of commercial platform operators (Google, Apple, etc.)?

Health data platform (non-commercial) founder

- Can you describe in a few words/key points why Open Humans was founded? / What is special about Open Humans (compared to other types of data platforms)?
- How can/should users of CHT be motivated to donate data for research purposes? (What specific approach do you take with Open Humans, which other approaches would be conceivable from your point of view, can this be generalised?)
- How can users of CHT be motivated to actively participate in research themselves (e.g. to actively collect more/specific data, propose research questions, analyse their own data, etc.)?
- What is the added value of more active participation in research, including citizen science (for research/for the participants)?

Medical IT experts

- Is it possible to identify specific fields of research (or even more specific diseases/disease groups) for which the use of CHT data is particularly relevant?
- Are there any regulatory, technical or other barriers to the use of the data? If so, please describe these by way of example!
- What opportunities and challenges (technical, legal, social) are there in combining clinical and CHT data specifically for research purposes?
- Which technical data protection concepts do you work with or are you developing these yourself? What particular challenges are there in relation to CHT data compared to clinical data?
- What form of consent do you work with? Could technical innovations ‘revolutionise’ consent procedures for CHT data donation in the future?
